# Age-dependent favorable visual recovery despite significant retinal atrophy in pediatric MOGAD: how much retina do you really need to see well?

**DOI:** 10.1186/s12974-021-02160-9

**Published:** 2021-05-29

**Authors:** Joachim Havla, Thivya Pakeerathan, Carolin Schwake, Jeffrey L. Bennett, Ingo Kleiter, Ana Felipe-Rucián, Stephanie C. Joachim, Amelie S. Lotz-Havla, Tania Kümpfel, Markus Krumbholz, Eva M. Wendel, Markus Reindl, Charlotte Thiels, Thomas Lücke, Kerstin Hellwig, Ralf Gold, Kevin Rostasy, Ilya Ayzenberg

**Affiliations:** 1 Institute of Clinical Neuroimmunology, LMU Hospital, Ludwig-Maximilians Universität München, Munich, Germany; 2 Data Integration for Future Medicine (DIFUTURE) Consortium, LMU Hospital, Ludwig-Maximilians Universität München, Munich, Germany; 3 Department of Neurology, St. Josef-Hospital, Ruhr-University Bochum, Bochum, Germany; 4 Departments of Neurology and Ophthalmology, Programs in Neuroscience and Immunology, University of Colorado Anschutz Medical Campus, Denver, USA; 5 Marianne-Strauß-Klinik, Behandlungszentrum Kempfenhausen für Multiple Sklerose Kranke, Berg, Germany; 6 Department of Pediatric Neurology, Vall d’Hebron Hospital, Universitat Autònoma de Barcelona, Barcelona, Spain; 7 Experimental Eye Research Institute, University Eye Hospital, Ruhr-University Bochum, Bochum, Germany; 8 Dr. von Hauner Children’s Hospital, LMU Hospital, Ludwig-Maximilians Universität München, Munich, Germany; 9 Department of Neurology & Stroke and Hertie Institute for Clinical Brain Research, University Hospital of Tübingen, Tübingen, Germany; 10 Department of Pediatric Neurology, Olgaspital Stuttgart, Stuttgart, Germany; 11 Clinical Department of Neurology, Medical University of Innsbruck, Innsbruck, Austria; 12 Department of Neuropaediatrics and Social Pediatrics, University Hospital of Pediatrics and Adolescent Medicine, Ruhr-University, Bochum, Germany; 13 Department of Pediatric Neurology, Children’s Hospital Datteln, University Witten/Herdecke, Witten, Germany; 14 Department of Neurology, Sechenov First Moscow State Medical University, Moscow, Russia

**Keywords:** Optical coherence tomography, Optic neuritis, Myelin oligodendrocyte glycoprotein IgG, MOGAD

## Abstract

**Background:**

To investigate age-related severity, patterns of retinal structural damage, and functional visual recovery in pediatric and adult cohorts of myelin oligodendrocyte glycoprotein antibody-associated disease (MOGAD) optic neuritis (ON).

**Methods:**

All MOGAD patients from the 5 participating centers were included. Patients with initial manifestation <18 years were included in the pediatric (MOGAD^ped^) cohort and patients with ≥18 years in the adult (MOGAD^adult^) cohort. For patients with MOGAD ON, examinations at least ≥6 months after ON onset were included in the analyses. Using spectral domain optical coherence tomography (SD-OCT), we acquired peripapillary retinal nerve fiber layer thickness (pRNFL) and volumes of combined ganglion cell and inner plexiform layer (GCIPL). High- and 2.5% low-contrast visual acuity (HCVA, LCVA) and visual-evoked potentials (VEP) were obtained.

**Results:**

Twenty MOGAD^ped^ (10.3±3.7 years, 30 MOGAD ON eyes) and 39 MOGAD^adult^ (34.9±11.6 years, 42 MOGAD ON eyes) patients were included. The average number of ON episodes per ON eye was similar in both groups (1.8±1.3 and 2.0±1.7). In both pediatric and adult MOGAD, ON led to pronounced neuroaxonal retinal atrophy (pRNFL: 63.1±18.7 and 64.3±22.9 μm; GCIPL: 0.42±0.09 and 0.44±0.13 mm^3^, respectively) and moderate delay of the VEP latencies (117.9±10.7 and 118.0±14.5 ms). In contrast, visual acuity was substantially better in children (HCVA: 51.4±9.3 vs. 35.0±20.6 raw letters, *p*=0.001; LCVA: 22.8±14.6 vs. 13.5±16.4, *p*=0.028). Complete visual recovery (HCVA-logMAR 0.0) occurred in 73.3% of MOGAD^ped^ and 31% MOGAD^adults^ ON eyes, while 3.3% and 31% demonstrated moderate to severe (logMAR > 0.5) visual impairment. Independent of retinal atrophy, age at ON onset significantly correlated with visual outcome.

**Conclusion:**

Pediatric MOGAD ON showed better visual recovery than adult MOGAD ON despite profound and almost identical neuroaxonal retinal atrophy. Age-related cortical neuroplasticity may account for the substantial discrepancy between structural changes and functional outcomes.

**Supplementary Information:**

The online version contains supplementary material available at 10.1186/s12974-021-02160-9.

## Introduction

Myelin oligodendrocyte glycoprotein antibody-associated disease (MOGAD) is a newly defined autoimmune disorder of the central nervous system (CNS) [1]. While immune responses targeting myelin oligodendrocyte glycoprotein (MOG) have been extensively studied in experimental autoimmune encephalomyelitis [1], the clinical relevance of MOG-immunoglobulin (Ig)G was only recently appreciated following the identification of autoantibodies targeting conformational epitopes of full-length MOG in humans [2]. MOGAD patients may develop any combination of monophasic or relapsing acute disseminated encephalomyelitis (ADEM), non-ADEM encephalitis, neuromyelitis optica spectrum disorder (NMOSD), optic neuritis (ON), transverse myelitis (TM), brainstem encephalitis, or rarely multiple sclerosis (MS)-like demyelinating disease [3–5]. Clinical manifestations seem to be age-dependent: children < 10 years are more likely to develop an ADEM phenotype, while those ≥10 years are more likely to present with an NMOSD- or MS-like phenotype similar to adults [6]. In both pediatric and adult patients, isolated, bilateral, or recurrent ON are common clinical presentations [3, 5, 7, 8]. High levels of MOG expression and enhanced blood-brain barrier permeability in the optic nerve may explain its frequent involvement [9, 10]. Although the histopathology of cerebral lesions in MOGAD patients demonstrates relative preservation of axonal structures, severe visual impairment or functional blindness have been reported in >30% of MOGAD^adults^ ON patients, and OCT studies repeatedly demonstrated profound axonal degeneration with loss of retinal ganglion cells [11–14]. Although MOG-IgG antibodies have been frequently reported in children with ON, there are only a few studies investigating retinal changes and visual outcomes in this population [5, 15–17]. Moreover, there are no studies comparing ON course and outcome in children and adults with MOGAD. The latter is especially interesting as pediatric MS and MOGAD patients generally demonstrate better relapse recovery than adults [18–22]. Here, we compared the outcomes of MOGAD^ped^ and MOGAD^adult^ ON including age-related (1) severity and patterns of retinal structural damage and (2) functional visual recovery.

## Subjects and methods

### Study population

We conducted an analysis of prospectively collected data from MOGAD^ped^ and MOGAD^adult^ patients with or without ON. The inclusion criteria included the acute presentation of demyelinating disease, MOG-IgG seropositivity, availability of OCT, visual acuity data, and clinical information. If one or multiple ON events were known, visits could be considered at least 6 months after the last ON. During the study period (2018–2020), all patients tested positive for MOG-IgG were included who met the inclusion criteria and who were seen at five academic university centers specialized in neuroimmunological diseases (Department of Pediatric Neurology, Children’s Hospital Datteln, University Witten/Herdecke, Germany, *N*=9; Department of Neuropediatrics and Social Pediatrics University Hospital of Pediatrics and Adolescent Medicine, Ruhr-University Bochum, *N*=3; Neurology Department, St. Josef Hospital Bochum, Bochum, Germany, *N*=16; Institute of Clinical Neuroimmunology, LMU Hospital, Munich, Germany, *N*=29, and Institute of Pediatrics, University Hospital Vall d’Hebron, Barcelona, Spain, *N*=2) [total *N*=59]. Five patients were excluded due to incomplete examination data (*n*=2) or an acute ON at the time of examination (*n*=3) (see supplementary Figure 3). A part of the MOGAD cohort of the Institute of Clinical Neuroimmunology (13/29 patients) has already been published as a subset of other cohort analyses [12, 13]. Written informed consent was obtained from all patients participating in the study. The local ethics committees approved the study protocol in accordance with the Declaration of Helsinki (1964) in its currently applicable version. Two groups of patients were evaluated depending on the age at disease manifestation: group 1 with 20 MOG-IgG-positive children with initial manifestation < 18 years (MOGAD^ped^) and group 2 with 39 MOG-IgG-positive adult patients with initial manifestation ≥ 18 years (MOGAD^adult^). Demographic (gender and age at initial manifestation) and clinical (disease duration, number and side of clinical ON episodes) data were collected for all patients. For the detection of MOG-IgG, serum samples were analyzed during the initial workup at least once by established cell-based assays at the discretion of each center using the laboratory’s cutoffs (MOG IFT, EUROIMMUN, Laboratory Stöcker, Germany; Reindl Lab, Medical University of Innsbruck, Innsbruck, Austria; Meinl Lab, LMU Hospital, Munich, Germany) [1, 2, 23].

### Optical coherence tomography (OCT) and visual acuity

All centers used spectral-domain optical coherence tomography (SD-OCT, SPECTRALIS, Heidelberg Engineering, Heidelberg, Germany) with automatic real-time (ART) averaging. A scan around the optic nerve with an activated eye tracker (12°, 3.5 mm ring, 50≤ ART ≤100) and a macular volume scan (20° × 20°, 25 vertical B-scans, 20 ≤ ART ≤ 49) were performed as cylinders of 3 mm diameter around the fovea based on local protocols. The thickness of the peripapillary retinal nerve fiber layer (pRNFL) and the volumes of the macular retinal nerve fiber (mRNFL), the combined ganglion cell and inner plexiform layer (GCIPL), the inner nuclear layer (INL), the combined outer plexiform layer and outer nuclear layer (OPONL), and the total macular volume (TMV) were analyzed. The segmentation of all layers was performed semi-automatically using software from the SD-OCT manufacturer (Eye Explorer 1.9.10.0 with viewing module 6.3.4.0, Heidelberg Engineering, Heidelberg, Germany). Experienced evaluators carefully checked all scans for sufficient quality and segmentation errors and corrected them if necessary. The SD-OCT data in this study are analyzed and reported according to the recommendations of APOSTEL and OSCAR-IB [24, 25].

**Fig. 1:**
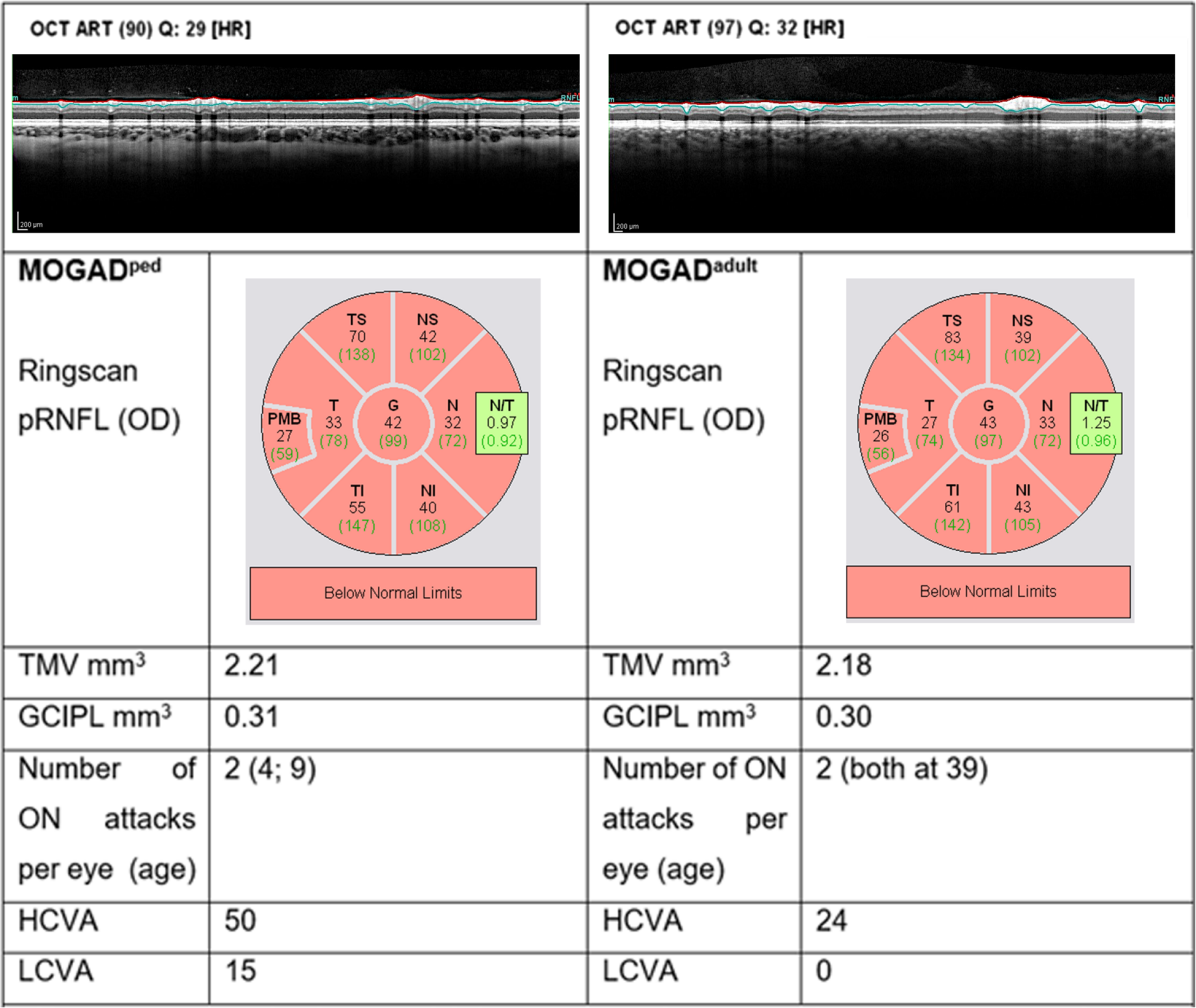
Representative pediatric and adult retinal OCT scans after recurrent MOGAD-ON. Despite almost identical neuroaxonal retinal atrophy, functional vision is notably better in children than adults. Abbreviations: *OD* oculus dexter, *G* global pRNFL, *N* nasal pRNFL, *I* inferior pRNFL, *T* temporal pRNFL, *PMB* paillo-macular-bundle, *TMV* total macular volume, *GCIPL* combined ganglion cell and inner plexiform layer

In addition, at the time of OCT examination, habitually corrected high-contrast and low-contrast monocular visual acuity (VA) was acquired using high contrast and 2.5% low-contrast Sloan letter charts placed in a retro-illuminated light box at 2 m distance. Each chart consists of 14 lines with 5 letters per line that are standardized with equal difficulty per line and equal spacing between the lines. The total number of correct letters identified on each chart was tested to determine high- and low-contrast VA (HCVA, LCVA; maximum, 70 letters). Characteristic pRNFL scans in a pediatric and an adult MOGAD patient are shown in Fig. 1.

### Visual evoked potentials (VEP)

VEP data (Keypoint.net, Neurolite Software, Natus, Switzerland) were collected from occipital midline referred to a mid-frontal electrode according to the International Society for Clinical Electrophysiology of Vision standards. Pattern reversal VEP was produced by high-contrast, black and white checks. Each check has the size of 171.6 arc minute. The examination was performed in a dark room in a 1-m distance. P100 latency and the P100-P125 amplitude were collected. All VEP examinations were performed in Bochum (data available for 15 MOGAD^ped^ and 12 MOGAD^adult^ patients).

### Statistical methods

Clinical data, OCT, VEP, and VA results were compared between MOGAD^ped^ and MOGAD^adult^ patients. The mean and standard deviation were calculated for continuous variables, frequency, and proportion for categorical variables. The non-parametric Mann-Whitney *U* test and chi-square test were used to compare two independent groups. Statistical significance was defined as *p* < 0.05.

SD-OCT data, VEP data, and HCVA/LCVA in the eyes with and without ON were analyzed within and between the MOGAD^ped^ and MOGAD^adult^ cohorts using generalized estimating equation models (GEE) to account for within-patient inter-eye correlation. The correlation matrix parameter was set to “exchangeable.”

Further, we performed a Spearman correlation to identify the possible factors determining the visual outcome in MOGAD ON. Age at ON, number of ON episodes per eye, the extent of retinal degeneration (pRNFL and GCIPL thickness), and VEP P100 latency were included in the analysis. For cases of recurrent ON, we calculated an average age at ON onset. Due to a relatively small sample size, both groups were pooled. All factors that significantly correlated with visual outcome were included in GEE analysis. Data were analyzed with SPSS version 26 (IBM SPSS Statistics).

## Results

### Cohort description

We enrolled 20 MOG-IgG-positive children (MOGAD^ped^ patients, female:male 13:7, mean age 10.3±3.7 years) and 39 MOG-IgG-positive adults (MOGAD^adult^ patients, female: male 20:19, mean age 34.9±11.6 years). From the medical history, 2 patients had no ON, 6 had unilateral ON, and 12 had bilateral ON in the MOGAD^ped^ patient cohort (total 30 ON affected eyes). Accordingly, in the MOGAD^adult^ patient cohort, 15 had no ON, 6 had unilateral ON, and 18 had bilateral ON (total 42 ON affected eyes). The main clinical data of all patients are described in Table 1. Eight of 20 MOG-IgG-positive children were diagnosed with recurrent NMOSD, 6 children with recurrent ON (rON), 3 children with ADEM + rON, and 3 children with encephalomyelitis. Three of 39 MOG-IgG-positive adults were diagnosed with monophasic NMOSD, 12 with recurrent NMOSD, 14 with encephalomyelitis, 9 adults with rON, and 1 adult with ADEM. There were no differences in gender, disease duration, or number of previous ON episodes between the two groups. Eighteen (94.7%) of the MOGAD^ped^ patients were on long-term immunotherapy at the time of SD-OCT (8 monotherapy with oral prednisone, 5 intravenous long-term immunoglobulin/subcutaneous immunoglobulin (IVIG/SCIG), 3 glatiramer acetate, 1 rituximab, 1 azathioprine, 1 oral prednisone as an add-on therapy) compared with 30 (76.9%) of MOGAD^adult^ patients (12 azathioprine, 7 rituximab, 1 oral prednisone as an add-on therapy, 3 methotrexate, 2 tocilizumab, 2 teriflunomide, 2 glatiramer acetate, 1 monotherapy with oral prednisone, and 1 long-term IVIG).

**Table 1:** Demographic and main clinical characteristics of pediatric and adult cohorts

	MOGAD^ped^ (*n*=20)	MOGAD^adult^ (*n*=39)	*p*
Age at initial manifestation, median (range)	10.5 (4–15)	35.0 (19–64)	**<0.001**
Females, *n* (%)	13 (65.0%)	20 (51.3%)	0.157
Ethnicity	20 Caucasians	36 Caucasians 3 Asians	0.081
Disease duration (years), median (range)	4.5 (0–22)	6.5 (0–24)	0.084
Patients with a clinical history of ON, *n* (%)	18 (90.0%)	24 (61.5%)	**0.001**
Patients with unilateral ON, *n* (%)	6 (30.0%)	6 (15.4%)	0.062
Patients with bilateral ON, *n* (%)	12 (60.0%)	18 (46.2%)	0.154
Patients with a simultaneous bilateral ON, *n* (%)	8 (40.0%)	14 (35.9%)	0.663
Total ON eyes, *n* (%)	30 (75.0%)	42 (53.8%)	**0.029**
Number of ON episodes per eye, mean (SD)	1.8 (1.3)	2.0 (1.7)	0.864
Time between ON and examination in months, median (range)	7 (6–129)	10 (6–155)	0.2

*Abbreviations*: *MOGAD* myelin oligodendrocyte glycoprotein antibody-associated disease, *ON* optic neuritis, *SD* standard deviation

### Pediatric and adult MOGAD ON cause profound neuroaxonal retinal atrophy

The main SD-OCT results are shown in Table 2 (for more detailed results, see supplementary Table 3 and supplementary Figure 4). The thickness of pRNFL was significantly reduced in MOGAD^ped^- and MOGAD^adult^-ON eyes compared to non-ON eyes globally as well as in all segments (pRNFL S, pRNFL I, pRNFL T, pRNFL N, pRNFL PMB); the pRNFL N/T ratio remained unchanged (see Fig. 1 for illustrating OCT images). The total macular volume (TMV), as well as combined GCIPL volume, were also significantly decreased in ON eyes of both pediatric and adult patients compared to non-ON eyes. In contrast, the volume of the inner nuclear layer (INL) was increased in ON eyes of both groups.

**Table 2:** Comparison of OCT and VEP measures as well as visual acuity between MOGAD-ON and MOGAD-NON eyes in pediatric and adult MOGAD patients

Parameter	Pediatric patients		Adult patients		Pediatric patients	Adult patients	MOGAD-ON eyes	MOGAD-NON eyes
	MOGAD-ON eyes (30 eyes, mean± SD)	MOGAD-NON eyes (10 eyes, mean± SD)	MOGAD-ON eyes (42 eyes, mean± SD)	MOGAD-NON eyes (36 eyes, mean± SD)	MOGAD-ON vs. MOGAD-NON eyes, *p*	MOGAD-ON vs. MOGAD-NON eyes, *p*	Pediatric vs. adult patients, *p*	Pediatric vs. adult patients, *p*
pRNFL G	63.12 ± 18.74	90.30 ± 13.40	64.26 ± 22.85	96.64 ± 20.67	**< 0.001**	**0.001**	0.997	0.292
TMV	2.19 ± 0.11	2.30 ± 0.13	2.22 ± 0.12	2.31 ± 0.11	**0.021**	**0.048**	0.484	0.718
mRNFL	0.12 ± 0.02	0.14 ± 0.01	0.13 ± 0.02	0.14 ± 0.03	**< 0.001**	0.537	**0.012**	0.278
mGCIPL	0.42 ± 0.09	0.57 ± 0.08	0.44 ± 0.13	0.57 ± 0.08	**< 0.001**	**0.012**	0.555	0.853
mINL	0.28 ± 0.03	0.26 ± 0.02	0.29 ± 0.04	0.26 ± 0.02	**0.001**	**0.017**	0.793	0.994
mOPONL	0.77 ± 0.07	0.74 ± 0.08	0.77 ± 0.05	0.75 ± 0.05	0.307	0.949	0.779	0.589
VEP P100 latency	117.86 ± 10.67	112.44 ± 9.59	117.99 ± 14.51	118.15 ± 9.84	0.324	0.790	0.946	0.115
VEP amplitude	10.57 ± 6.12	8.52 ± 4.47	5.76 ± 2.79	7.61 ± 5.21	0.235	**0.049**	–	–
HC VA	51.36 ± 9.33	55.60 ± 8.88	34.97 ± 20.57	52.03 ± 8.67	0.245	**0.002**	**< 0.0001**	0.325
2.5% LC VA	22.83 ± 14.62	25.60 ± 13.94	13.54 ± 16.44	29.52 ± 13.89	0.963	**0.017**	**0.028**	0.451

GEE analysis: *p* value: significant results *p* < 0.05 are indicated in bold

*Abbreviations*: *MOGAD-ON* eyes with a history of ON, *MOGAD-NON* eyes without a history of ON, *pRNFL* peripapillary retinal nerve fiber layer (G global), *TMV* total macular volume, *mRNFL* macular RNFL, *mGCIPL* macular ganglion cell and inner plexiform layer, *mINL* macular inner nuclear layer, *mOPONL* macular outer plexiform and outer nuclear layer, *HC* high-contrast, *LC* low-contrast, VA visual acuity, *pRNFL* thickness in μm, macular volumes in mm^3^, *VEP P100* latency in ms, *VEP* amplitude in uV, *VA* number of correctly stated letters

Comparing MOGAD^ped^-ON and MOGAD^adult^-ON eyes, we found no differences in pRNFL thickness or volumes of the macular GCIPL, INL, and OPONL, while macular RNFL was significantly lower in MOGAD^ped^-ON eyes compared to MOGAD^adult^-ON eyes. Macular microcysts were observed in 10% of MOGAD^ped^-ON and 7.7% of MOGAD^adult^-ON eyes.

### Peripapillary retinal atrophy patterns are similar independently of age

There was no difference in the pattern of neuroaxonal retinal atrophy between MOGAD^ped^ and MOGAD^adult^ ON eyes (Table 3, supplementary results). MOGAD ON eyes showed no temporal predominance of pRNFL atrophy after 1.8±1.3 vs. 2.0±1.7 ON episodes per eye in children and adults respectively. The N/T ratio was comparable in affected eyes of pediatric and adult patients.

### Children demonstrate significantly better visual outcome after MOGAD ON

VA testing revealed significantly worse HCVA and LCVA in affected MOGAD^adult^-ON eyes (Table 2, supplementary Figure 4). This was not the case for affected MOGAD^ped^-ON eyes. Complete visual recovery (defined as HCVA logMAR 0.0) occurred in 73.3% of MOGAD^ped^-ON eyes vs. 31.0% MOGAD^adult^-ON eyes. Only 3.3% of MOGAD^ped^-ON eyes vs. 31.0% of MOGAD^adult^-ON eyes showed moderate to severe (HCVA logMAR > 0.5) visual impairment. HCVA and LCVA were significantly worse in affected eyes of MOGAD^adult^ versus MOGAD^ped^ patients; there was no difference noted in unaffected non-ON eyes.

### VEP are not different in affected eyes in pediatric and adult MOGAD patients

VEP latencies were only moderately prolonged and showed no significant differences between affected and unaffected eyes in both cohorts (Table 2). Interestingly, the VEP latencies were also almost identical in affected eyes in MOGAD^ped^ and MOGAD^adult^ patients. Significant reduction of the VEP amplitudes was observed in the affected eyes of MOGAD^adult^ patients only. VEP amplitude varies with age and was not compared between MOGAD^ped^ and MOGAD^adult^ patients.

### Age at onset correlates with visual outcome in MOGAD ON

**Fig. 2:**
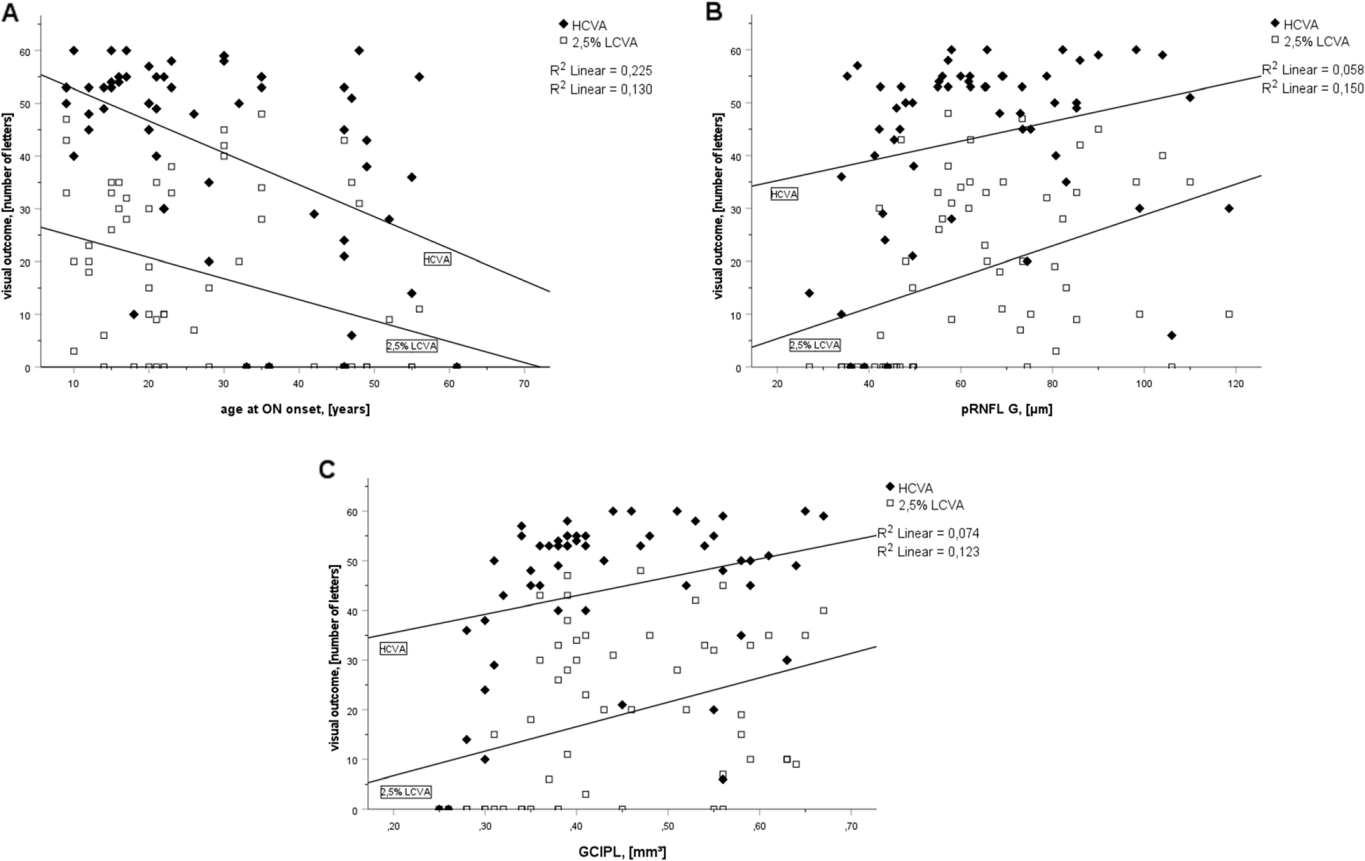
Scatterplots of age at ON onset (**a**), pRNFL G thickness (**b**), and GCIPL volume (**c**) against visual outcome (HCVA and 2.5% LCVA). HCVA correlated modestly with neuroaxonal retinal atrophy and more strongly with age at ON onset, whereas LCVA correlated moderately with both parameters

We next evaluated the effects of the number of previous ON attacks, age at ON onset, extent of neuroaxonal retinal atrophy and optic nerve signal transmission (P100 latency) on the HCVA, and LCVA in the entire study population (Fig. 2 and supplementary Figure 5). Neither the number of previous ON nor P100 latency correlated with HCVA or LCVA. Neuroaxonal retinal atrophy weakly correlated with HCVA (rho=0.311 CI95% 0.080–0.510, *p*=0.014 [pRNFL] and rho=0.282 CI95% 0.049–0.486, *p*=0.036 [GCIPL]) and more closely with a LCVA reduction (rho=0.498 CI95% 0.288 0.662, *p*<0.001 [pRNFL] and rho=0.448 CI95% 0.230 0.623, *p*=0.001 [GCIPL]) (Fig. 2). We observed a stronger correlation with the age at ON onset (rho=−0.565 CI95% −0.713–0.368, *p*<0.001 [HCVA] and rho=−0.460 CI95% −0.632–0.244, *p*<0.001 [LCVA]).

## Discussion

In this study, we were able to demonstrate a significantly better visual recovery after ON in pediatric versus adult MOGAD patients. Interestingly, substantial differences in the functional outcome in these two groups were independent of structural neuroaxonal retinal atrophy and signal transmission in the optic nerve, so that other age-dependent mechanisms are likely to be responsible. Our results thus add nicely to the recently published work on clinical features and risk of relapse in children and adults with MOGAD [22]. In this study, overall better remission of general relapses was reported in MOGAD^ped^ compared to MOGAD^adult^ patients, although the underlying mechanisms remained unclear [22].

On a structural level, we clearly observed both pRNFL and GCIPL atrophy in the affected eye compared with the unaffected eyes in MOGAD^ped^ and MOGAD^adult^ patients, indicating combined neuroaxonal retinal atrophy. The extent of degeneration was comparable across both cohorts and in line with recent studies. Previously, we and others have shown that pRNFL parameters, TMV, mRNFL, mGCL, and mIPL are significantly reduced in MOGAD^adult^-ON eyes compared to healthy controls [11–13]. There is little data on structural changes after MOGAD ON in children. Two other groups have reported significant neuroaxonal retinal atrophy following MOGAD^ped^-ON with an average and median pRNFL thickness of 68.7 ± 12.6 μm and 58 μm, respectively [26, 27]. These findings are comparable to our pRNFL measures (mean 63.1±18.7 μm). Data on GCIPL thickness are only available from one Chinese cohort. Similar to our study, the combined GCIPL volume in MOGAD^ped^-ON was significantly reduced [26].

The volume of the INL was increased in ON eyes of both MOGAD^ped^ and MOGAD^adult^ patients. It has been suggested that the INL may serve as a biomarker for inflammatory processes, since an increase in INL volume is associated with MS-ON and risk for clinical relapse [28]. However, the meaning of the INL volume change in MOGAD remains uncertain and may occur in compensation to neuroaxonal retinal atrophy. We could not confirm an association between INL volume and relapse rate in our cohort (data not shown). Microcystic macular edema (MME) in the INL has been documented in MOGAD ON [12, 13]. We observed INL-MME in 10% vs. 7.7% of MOGAD^ped^- and MOGAD^adult^-ON, but not in unaffected eyes. In contrast to MS, we did not observe predominant temporal atrophy or any other specific pattern of RNFL loss. We found a high rate of RNFL atrophy in all optic nerve head segments [29].

MOGAD ON has been reported, in general, to have a more favorable visual prognosis when compared to AQP-4 IgG-seropositive NMOSD ON [30–35]. Study results, however, varied significantly, probably due in part to MOGAD clinical heterogeneity [11, 12, 27, 30–32, 34, 36–38]. Our adult MOGAD cohort, being partly included in previous multicenter studies, showed significant visual impairment in a relatively high proportion of patients after MOGAD ON [12, 13]. Nevertheless, other studies also reported functional blindness in MOGAD ON patients, with a VA below logMAR 1.0 in 13% of MOGAD ON eyes [11, 13]. In contrast, we observed a very good functional recovery in the pediatric cohort despite similar levels of neuroaxonal retinal atrophy. Interestingly, another study also demonstrated a more favorable functional outcome in MOGAD ON compared to AQP4-IgG seropositive NMOSD ON despite similar structural pRNFL degeneration [37]. HCVA in our MOGAD^ped^-ON cohort was similar to that reported by Wan et al. (complete recovery in 85% of affected eyes [*n*=59, mean age 12.6, range 3.9–18.8]) [36]. In contrast, Eyre et al. showed that only 65% of children from the mixed cohort recovered completely; microstructural damage of the retina was the only factor correlating with visual recovery [38].

The association of better visual recovery with younger age at ON onset is a central finding of this study. The better visual recovery in younger patients was independent of the number of previous ON attacks per eye, microstructural retinal changes, or optic nerve signal transmission. This is generally consistent with previous observations of better visual recovery in younger patients with AQP4-abs-positive NMOSD-ON and MS-ON, although these studies did not control for the number of previous ON episodes, optic nerve signal transmission, and neuroaxonal retinal atrophy [21, 33, 34]. In a recently published analysis of the relapse recovery of children versus adults with MS, the probability of incomplete recovery increased 1.33-fold with each decade of life [39].

We propose that an active age-dependent neuroplasticity of the visual system, most likely at a cortical level, may explain our findings. First, there is evidence for the existence of immature neural visual circuits in children enabling further development of visual acuity in childhood and adolescence [40]. Indeed, the HCVA of healthy children up to the age of 8–10 years is inferior to that of healthy adults, and the age of 6–8 years is widely accepted as a “sensitive period” for the development of amblyopia [40–42]. Second, animal studies have shown that contrast sensitivity is less promoted by retinal integrity than by the maturation of visual circuits [43]. In children, contrast sensitivity matures between the age of 8 and 19 [41]. Accordingly, visual system maturation is likely to be ongoing in our MOGAD^ped^ cohort (mean age 10.3±3.7 years). Indeed, we observed only minimal differences in HCVA and LCVA between affected and non-affected^-^ MOGAD^ped^-ON eyes, regardless of the profound neuroaxonal retinal atrophy. Third, a large functional magnetic resonance imaging (fMRI) study demonstrated a clear association between fMRI activity in the lateral occipital cortex (as a marker of early adaptive neuroplasticity) and visual outcome in young adults (mean age 32 years) with acute ON. Interestingly, the fMRI activity was the strongest predictor of the visual outcome in this study and was also independent of other structural or electrophysiological parameters at baseline or 12 months follow-up [44].

Given the heterogeneity of MOGAD, we cannot exclude that age- or disease-dependent differences in the MOGAD autoimmunity contribute to the noted differences in ON recovery. Indeed, the role of MOG-IgG in disease pathogenesis is not clear, and the titer and epitope specificity of MOG-IgG may drive distinct effects during injury and recovery [34, 35]. Additionally, other cellular mechanisms, including T cell and microglial response, need to be further characterized [2, 14]. Nevertheless, in our cohorts, these immunologic variables did not impact structural or electrophysiologic metrics.

Our study has several limitations. Due to the limited dataset, the extent of visual impairment before the onset of ON and at nadir remains unknown and cannot be compared between the groups. Visual acuity was corrected habitually, and there is no visual field or color vision data. In addition, our study lacks MRI data on the extent and volume of optic nerve lesions and accompanying lesions in the post-geniculate visual pathways or occipital cortex. A prospective study combining longitudinal clinical data, OCT and neuro-imaging, visual field data, electrophysiology, and fMRI would be important to confirm our findings. Furthermore, studies in MS or aquaporin-4-IgG-positive NMOSD are needed to clarify if the demonstrated age-dependent VA improvement is universal or disease-specific.

## Conclusion

In summary, a comparison of pediatric and adult MOGAD cohorts demonstrates age-dependent effects on visual recovery after ON. Despite almost identical neuroaxonal retinal atrophy, functional vision is notably better in children than in adults. Age-dependent cortical neuroplasticity seems to be the most plausible mechanism explaining this dissociation. Future studies, combining precise analysis of the anterior and posterior visual system, are needed to confirm the leading role of central neuroplasticity in determining visual recovery after MOGAD-ON. Identification of a functionally relevant cutoff age for the visual recovery in MOGAD-ON could be of high relevance for the prognosis and care.

## Supplementary Information

**Additional file 1: **Supplementary Figure 3: Flow chart of patients included in the study. During the study period 64 MOGAD patients were identified in participating centers. Five patients were excluded due to incomplete examination data (*n*=2) or an acute ON at the time of examination (*n*=3). Depending on the age of manifestation patients were divided into 2 groups: group (1) 20 MOG-IgG-positive patients with initial manifestation < 18 years (MOGAD^ped^) and group (2) 39 MOG-IgG-positive patients with initial manifestation ≥ 18 years (MOGAD^adult^). Supplementary Figure 4: Beeswarm plots showing the distribution of pRNFL G thickness (A), GCIPL volume (B), HCVA (C) and 2,5% LCVA (D) in MOGAD^ped^-ON and MOGAD^adult^-ON. Despite profound neuroaxonal retinal atrophy in both groups, MOGAD^adult^-ON eyes showed significantly worse visual outcome (HCVA and 2,5% LCVA) in comparison to MOGAD^ped^-ON. Supplementary Figure 5: Scatterplots of VEP P100 latency (A) and number of previous ON (B) against visual outcome (HCVA and 2,5% LCVA). Neither the number of previous ON nor P100-latency correlated with HCVA (rho=0.030 CI95% -0.288 -0.152, p=0.840 [number of previous ON] and rho=-0.028 CI95% -0.354 -0.290, p=0.851 [VEP P100 latency]) or LCVA (rho=-0.237 CI95% -0.422 -0.106, p=0.104 [number of previous ON] and rho=-0.263 CI95% -0.503 -0.078, p=0.071 [VEP P100 latency]). Supplementary Table 3: Demographic and main clinical characteristics of pediatric and adult cohorts with ON history (MOGAD-ON). Abbreviations: MOGAD myelin oligodendrocyte glycoprotein-antibody-associated disease, ON optic neuritis, SD standard deviation. Supplementary Table 4. Comparison of all OCT and VEP measures as well as visual acuity between in ON- and non-ON-eyes in pediatric and adult MOGAD patients. Abbreviations: MOGAD-ON eyes with a history of ON, MOGAD-NON eyes without history of ON, pRNFL peripapillary retinal nerve fibre layer (G global, S superior, l inferior, T temporal, N nasal, PMB papillomacular bundle, N/T nasal/temporal ratio), TMV total macular volume, mRNFL macular RNFL, mGCIPL macular ganglion cell and inner plexiform layer, mINL macular inner nuclear layer, mOPONL macular outer plexiform and outer nuclear layer, HCVA high-contrast, LCVA low-contrast visusal acuity. pRNFL thickness in μm and macular volumes in mm^3^, VEP P100 latency in ms, VEP amplitude in uV, VA in number of correctly stated letters. GEE analysis: B regression coefficient, *p*-value: significant results *p* < 0.05 are indicated in bold letters.

## Data Availability

The data that support the findings of this study are available from the corresponding author upon reasonable request.
