# Supplementary material for: Age-dependent favorable visual recovery despite significant retinal atrophy in pediatric MOGAD: how much retina do you really need to see well?
Source: J Neuroinflammation. 2021 May 29;18:121. doi: 10.1186/s12974-021-02160-9 (PMC8164737; doi:10.1186/s12974-021-02160-9)
Supplement: Supplementary file 1 — Additional file 1: Supplementary Figure 3: Flow chart of patients included in the study. During the study period 64 MOGAD patients were identified in participating centers. Five patients were excluded due to incomplete examination data (n=2) or an acute ON at the time of examination (n=3). Depending on the age of manifestation patients were divided into 2 groups: group (1) 20 MOG-IgG-positive patients with initial manifestation < 18 years (MOGADped) and group (2) 39 MOG-IgG-positive patients with initial manifestation ≥ 18 years (MOGADadult). Supplementary Figure 4: Beeswarm plots showing the distribution of pRNFL G thickness (A), GCIPL volume (B), HCVA (C) and 2,5% LCVA (D) in MOGADped-ON and MOGADadult-ON. Despite profound neuroaxonal retinal atrophy in both groups, MOGADadult-ON eyes showed significantly worse visual outcome (HCVA and 2,5% LCVA) in comparison to MOGADped-ON. Supplementary Figure 5: Scatterplots of VEP P100 latency (A) and number of previous ON (B) against visual outcome (HCVA and 2,5% LCVA). Neither the number of previous ON nor P100-latency correlated with HCVA (rho=0.030 CI95% -0.288 -0.152, p=0.840 [number of previous ON] and rho=-0.028 CI95% -0.354 -0.290, p=0.851 [VEP P100 latency]) or LCVA (rho=-0.237 CI95% -0.422 -0.106, p=0.104 [number of previous ON] and rho=-0.263 CI95% -0.503 -0.078, p=0.071 [VEP P100 latency]). Supplementary Table 3: Demographic and main clinical characteristics of pediatric and adult cohorts with ON history (MOGAD-ON). Abbreviations: MOGAD myelin oligodendrocyte glycoprotein-antibody-associated disease, ON optic neuritis, SD standard deviation. Supplementary Table 4. Comparison of all OCT and VEP measures as well as visual acuity between in ON- and non-ON-eyes in pediatric and adult MOGAD patients. Abbreviations: MOGAD-ON eyes with a history of ON, MOGAD-NON eyes without history of ON, pRNFL peripapillary retinal nerve fibre layer (G global, S superior, l inferior, T temporal, N nasal, PMB papillomacular bundle, N/T [file 12974_2021_2160_MOESM1_ESM.pdf]

Supplementary Material

RESEARCH

Age-dependent favorable visual recovery despite significant retinal atrophy in pediatric MOGAD: how much retina do you really need to see well?

Running head:

Age-dependent visual recovery in MOGAD-ON

Joachim Havla MD[1,2*§], Thivya Pakeerathan[3*], Carolin Schwake MD[3], Jeffrey L. Bennett MD, PhD[4], Ingo Kleiter MD[3,5], Ana Felipe-Rucián MD[6], Stephanie C. Joachim MD[7], Amelie S. Lotz-Havla MD[8], Tania Kümpfel MD[1], Markus Krumbholz MD[9], Eva M. Wendel MD[10], Markus Reindl MD[11], Charlotte Thiels MD[12], Thomas Lücke MD[12], Kerstin Hellwig MD[3], Ralf Gold MD[3], Kevin Rostasy MD[13#] and Ilya Ayzenberg MD[3,14#§]

[*,#] *These authors contributed equally to the manuscript*

[1] Institute of Clinical Neuroimmunology, LMU Hospital, Ludwig-Maximilians Universität München, Munich, Germany

[2] Data Integration for Future Medicine (DIFUTURE) consortium, LMU Hospital, Ludwig-Maximilians Universität München, Munich, Germany

[3] Department of Neurology, St. Josef-Hospital, Ruhr-University Bochum, Bochum, Germany.

[4]Departments of Neurology and Ophthalmology, Programs in Neuroscience and Immunology, University of Colorado Anschutz Medical Campus, Denver, CO, 80045, USA.

[5]Marianne-Strauß-Klinik, Behandlungszentrum Kempfenhausen für Multiple Sklerose Kranke, Berg, Germany

[6] Department of Paediatric Neurology, Universitat Autònoma de Barcelona, Vall d'Hebron Hospital, Barcelona, Spain

[7] Experimental Eye Research Institute, University Eye Hospital, Ruhr-University Bochum, Bochum, Germany.

[8] Dr. von Hauner Children's Hospital, LMU Hospital, Ludwig-Maximilians Universität München, Munich, Germany

[9] Department of Neurology & Stroke and Hertie Institute for Clinical Brain Research, University Hospital of Tübingen, Tübingen, Germany

[10] Olgaspital Stuttgart, Department of Pediatric Neurology, Stuttgart, Germany

[11] Clinical Department of Neurology, Medical University of Innsbruck, Innsbruck, Austria

[12] Department of Neuropaediatrics and Social Pediatrics, University Hospital of Pediatrics and Adolescent Medicine, Ruhr-University, Bochum, Germany

[13] Department of Paediatric Neurology, Children’s Hospital Datteln, University Witten/Herdecke, Germany

[14] Department of Neurology, Sechenov First Moscow State Medical University, Moscow, Russia

Keywords:

Optical coherence tomography, Optic neuritis, Myelin-oligodendrocyte-glycoprotein IgG, MOGAD

Supplementary material

Supplementary Figure 3: Flow chart of patients included in the study

During the study period 64 MOGAD patients were identified in participating centers. Five patients were excluded due to incomplete examination data (n=2) or an acute ON at the time of examination (n=3). Depending on the age of manifestation patients were divided into 2 groups: group (1) 20 MOG-IgG-patients with initial manifestation < 18 years (MOGAD^{ped}) and group (2) 39 MOG-IgG-positive patients with initial manifestation ≥ 18 years (MOGAD^{adult}).

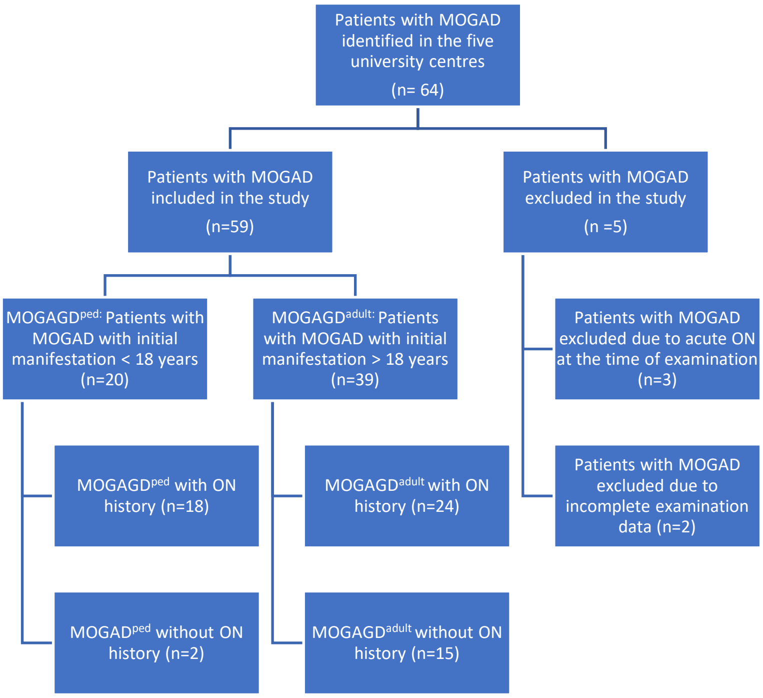

Supplementary Figure 4: Beeswarm plots showing the distribution of pRNFL G thickness (A), GCIPL volume (B), HCVA (C) and 2,5% LCVA (D) in MOGAD^{ped}-ON and MOGAD^{adult}-ON.

Despite profound neuroaxonal retinal atrophy in both groups, MOGAD^{adult}-ON eyes showed significantly worse visual outcome (HCVA and 2,5% LCVA) in comparison to MOGAD^{ped}-ON.

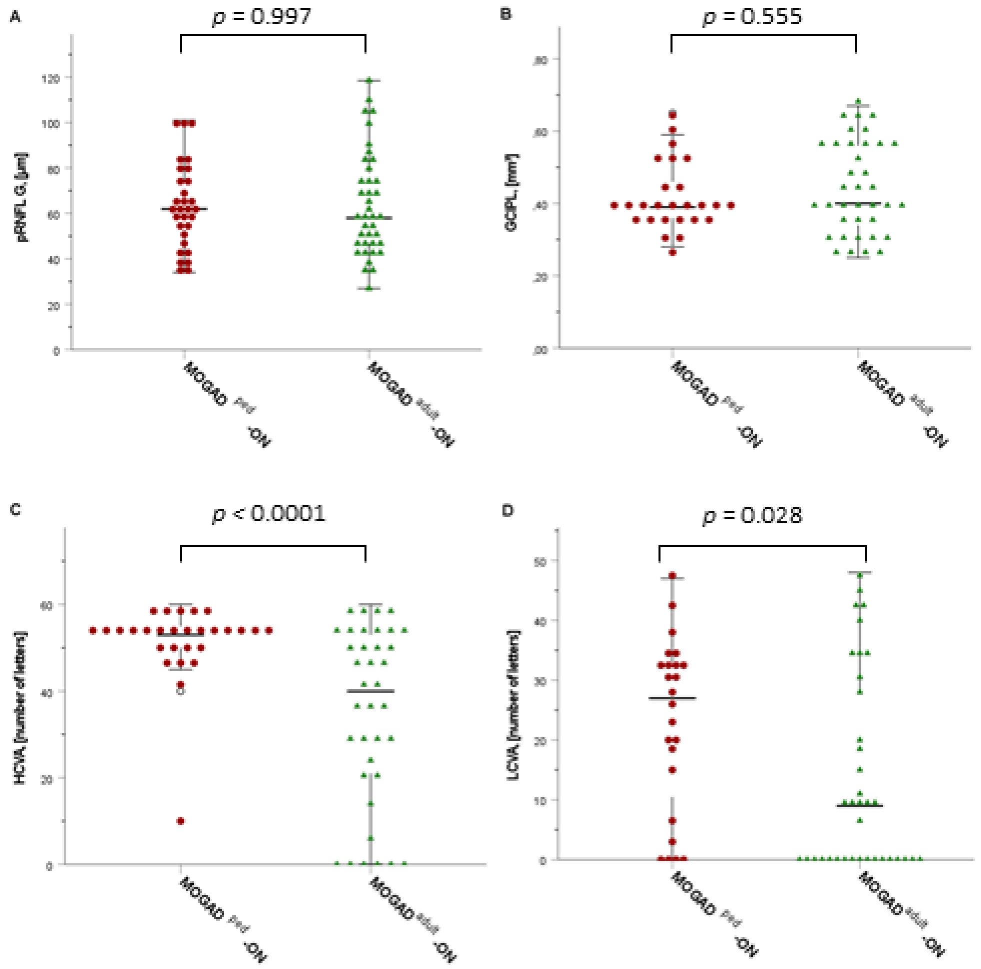

Supplementary Figure 5: Scatterplots of VEP P100 latency (A) and number of previous ON (B) against visual outcome (HCVA and 2,5% LCVA).

Neither the number of previous ON nor P100-latency correlated with HCVA (rho=0.030 CI95% -0.288 -0.152, p=0.840 [number of previous ON] and rho=-0.028 CI95% -0.354 -0.290, p=0.851 [VEP P100 latency]) or LCVA (rho=-0.237 CI95% -0.422 -0.106, p=0.104 [number of previous ON] and rho=-0.263 CI95% -0.503 -0.078, p=0.071 [VEP P100 latency]).

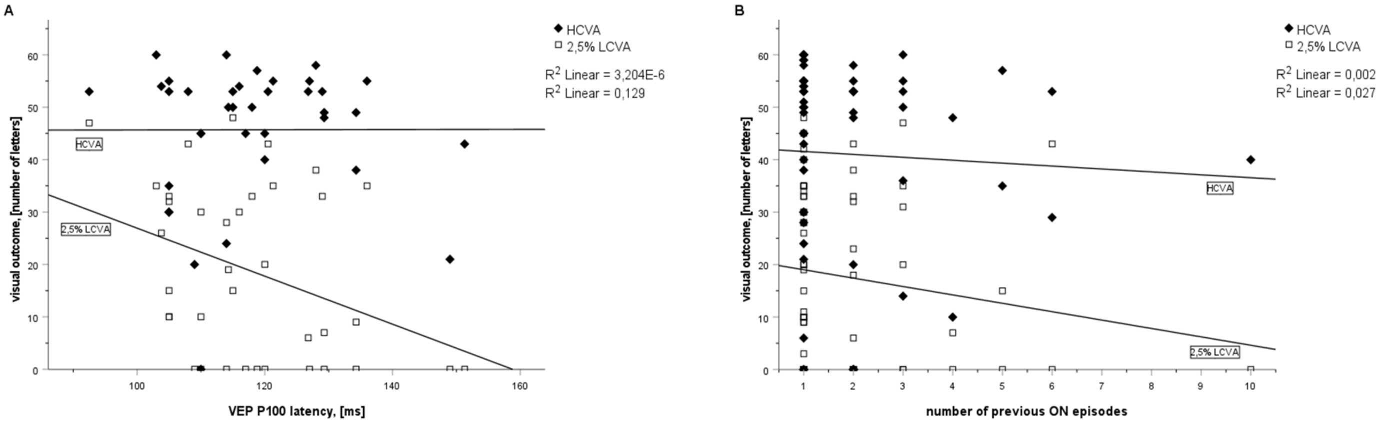

**Suppl. Table 3**	MOGAD^{ped}-ON (n = 18)	MOGAD^{adult}-ON (n = 24)	p
Age at initial manifestation, median (range)	10 (4-15)	33 (19-56)	<0.001
Females, n (%)	12 (66.7%)	20 (83.3%)	0.067
Ethnicity	18 Caucasian	22 Caucasian<br>2 Asian	0.151
Disease duration (in years), median (range)	3 (0-16)	7 (0-24)	0.280
Number of ON episodes per ON eye, mean (SD); median (range)	1.8 (1.3); 1 (5)	2.0 (1.7); 1 (9)	0.864
Time interval between examination and ON onset in months median (range)	7 (6-129)	10 (6-155)	0.2
ON eyes with moderate to severe visual impairment (HCVA logMAR > 0.5), n (%)	1 (3.3%)	13 (31.0%)	0.004

Supplementary Table 3: Demographic and main clinical characteristics of pediatric and adult cohorts with ON history (MOGAD-ON)

Abbreviations: MOGAD myelin oligodendrocyte glycoprotein-antibody-associated disease, ON optic neuritis, SD standard deviation

**Suppl. Table 4**	Pediatric patients		Adult patients		Pediatric patients MOGAD-ON vs. MOGAD-NON eyes		Adult patients MOGAD-ON vs. MOGAD-NON eyes		MOGAD-ON eyes Pediatric vs. adult patients		MOGAD-NON eyes Pediatric vs. adult patients	
	ON eyes (30 eyes, mean± SD)	non-ON eyes (10 eyes, mean± SD)	ON eyes (42 eyes, mean± SD)	non-ON eyes (36 eyes, mean± SD)	B	p	B	p	B	p	B	p
pRNFL G	63.12 ± 18.74	90.30 ± 13.40	64.26 ± 22.85	96.64 ± 20.67	**23.90**	**< 0.001**	**24.92**	**0.001**	0.02	0.997	-6.34	0.292
pRNFL S	78.90 ± 28.03	115.60 ± 18.46	82.00 ± 31.70	118.46 ± 35.66	**31.49**	**< 0.001**	**27.76**	**0.01**	-1.39	0.869	-2.86	0.754
pRNFL I	81.33 ± 26.94	114.00 ± 15.85	82.82 ± 33.35	119.56 ± 31.96	**28.28**	**0.001**	**25.34**	**0.021**	0.13	0.989	-5.56	0.464
pRNFL T	46.33 ± 19.75	64.80 ± 21.39	46.13 ± 18.13	69.06 ± 18.23	**16.68**	**0.029**	**19.32**	**0.001**	0.82	0.884	-4.26	0.594
pRNFL N	43.23 ± 12.82	66.80 ± 12.04	46.26 ± 17.83	76.46 ± 20.24	**21.67**	**< 0.001**	**24.23**	**< 0.001**	-2.28	0.595	-9.66	0.061
pRNFL PMB	35.70 ± 13.72	49.50 ± 12.75	38.41 ± 18.77	54.03 ± 18.49	**12.42**	**0.012**	**11.26**	**0.013**	-2.17	0.631	-4.53	0.408
pRNFL N/T ratio	1.05 ± 0.37	1.12 ± 0.37	1.08 ± 0.57	1.14 ± 0.32	0.037	0.771	0.01	0.927	-0.02	0.86	-0.03	0.835
TMV	2.19 ± 0.11	2.30 ± 0.13	2.22 ± 0.12	2.31 ± 0.11	**0.09**	**0.021**	**0.09**	**0.048**	-0.03	0.484	-0.02	0.718
mRNFL	0.12 ± 0.02	0.14 ± 0.01	0.13 ± 0.02	0.14 ± 0.03	**0.02**	**< 0.001**	0.01	0.537	**-0.01**	**0.012**	-0.01	0.278
mGCIPL	0.42 ± 0.09	0.57 ± 0.08	0.44 ± 0.13	0.57 ± 0.08	**0.15**	**< 0.001**	**0.10**	**0.012**	-0.02	0.555	0.01	0.853
mINL	0.28 ± 0.03	0.26 ± 0.02	0.29 ± 0.04	0.26 ± 0.02	**-0.03**	**0.001**	**- 0.022**	**0.017**	-0.003	0.793	-0.00005	0.994
mOPONL	0.77 ± 0.07	0.74 ± 0.08	0.77 ± 0.05	0.75 ± 0.05	-0.036	0.307	0.001	0.949	0.006	0.779	- 0.02	0.589
VEP P100 latency	117.86 ± 10.67	112.44 ± 9.59	117.99 ± 14.51	118.15 ± 9.84	-4.50	0.324	-1.49	0.790	-0.32	0.946	-5.71	0.115
VEP amplitude	10.57 ± 6.12	8.52 ± 4.47	5.76 ± 2.79	7.61 ± 5.21	-2.66	0.235	**2.78**	**0.049**	-		-	
HC VA	51.36 ± 9.33	55.60 ± 8.88	34.97 ± 20.57	52.03 ± 8.67	4.35	0.245	**17.03**	**0.002**	**16.36**	**< 0.0001**	3.57	0.325
2,5% LC VA	22.83 ± 14.62	25.60 ± 13.94	13.54 ± 16.44	29.52 ± 13.89	0.277	0.963	**12.56**	**0.017**	**10.36**	**0.028**	-3.92	0.451

Supplementary Table 4. Comparison of all OCT and VEP measures as well as visual acuity between in ON- and non-ON-eyes in pediatric and adult MOGAD patients

Abbreviations: MOGAD-ON eyes with a history of ON, MOGAD-NON eyes without history of ON, pRNFL peripapillary retinal nerve fibre layer (G global, S superior, I inferior, T temporal, N nasal, PMB papillomacular bundle, N/T nasal/temporal ratio), TMV total macular volume, mRNFL macular RNFL, mGCIPL macular ganglion cell and inner plexiform layer, mINL macular inner nuclear layer, mOPONL macular outer plexiform outer nuclear layer, HC high-contrast, LC low-contrast. pRNFL thickness are expressed in µm and macular volumes in mm³, VEP P100 latency in ms, VEP amplitude in uV, VA in number of correctly stated letters. GEE analysis: B regression coefficient, p-value: significant results p < 0.05 are indicated in bold letters.
